# Functional Connectivity-Derived Optimal Gestational-Age Cut Points for Fetal Brain Network Maturity

**DOI:** 10.3390/brainsci11070921

**Published:** 2021-07-12

**Authors:** Josepheen De Asis-Cruz, Scott Douglas Barnett, Jung-Hoon Kim, Catherine Limperopoulos

**Affiliations:** Developing Brain Institute, Children’s National, Washington, DC 20010, USA; jocruz@childrensnational.org (J.D.A.-C.); sbarnett2@childrensnational.org (S.D.B.); jkim9@childrensnational.org (J.-H.K.)

**Keywords:** resting-state, fetal connectome, graph theory, neurodevelopment, functional MRI, functional connectivity

## Abstract

The architecture of the human connectome changes with brain maturation. Pivotal to understanding these changes is defining developmental periods when transitions in network topology occur. Here, using 110 resting-state functional connectivity data sets from healthy fetuses between 19 and 40 gestational weeks, we estimated optimal gestational-age (GA) cut points for dichotomizing fetuses into ‘young’ and ‘old’ groups based on global network features. We computed the small-world index, normalized clustering and path length, global and local efficiency, and modularity from connectivity matrices comprised 200 regions and their corresponding pairwise connectivity. We modeled the effect of GA at scan on each metric using separate repeated-measures generalized estimating equations. Our modeling strategy involved stratifying fetuses into ‘young’ and ‘old’ based on the scan occurring before or after a selected GA (i.e., 28 to 33). We then used the quasi-likelihood independence criterion statistic to compare model fit between ‘old’ and ‘young’ cohorts and determine optimal cut points for each graph metric. Trends for all metrics, except for global efficiency, decreased with increasing gestational age. Optimal cut points fell within 30–31 weeks for all metrics coinciding with developmental events that include a shift from endogenous neuronal activity to sensory-driven cortical patterns.

## 1. Introduction

Recent advances in brain imaging have enabled researchers to investigate in vivo human brain development. Resting-state functional connectivity MRI (RS-fMRI), specifically, has allowed us to noninvasively probe developing functional connections across multiple brain networks simultaneously in fetuses [1,2]. Understanding neural circuitry formation in healthy fetuses is critical in recognizing early signs of functional alterations caused by genetic or environmental insults. The timing of developmental events can help differentiate aberrant from neurotypical processes. Thus, perinatal RS-fMRI aims to detect when functional networks emerge (e.g., appearance of visual and sensorimotor RSNs) and to identify when networks mature (e.g., become more similar to newborn architecture) [3,4,5,6].

While fetal imaging is a nascent field, findings over the past decade have begun to shed light on patterns of neural circuitry formation: connectivity follows a mediolateral, postero-anterior timing [4,7,8], occipital and sensorimotor circuits develop earlier than parietal connections [4], lateralization appears in superior temporal cortices in utero [3], and intra- and interhemispheric connectivity strength increase with maturity [9]. Age-related changes are not restricted to localized or regional connections. Systems-wide changes in network integration and segregation associated with brain maturation have been described using graph-theoretic techniques, a quantitative framework for describing complex networks such as the brain [10,11]. Studies have shown the emergence of small-world network topology as early as the second trimester and reduced segregation with advancing GA based on decreasing modularity, normalized clustering, and local efficiency [5,12,13]. While trends have been described, it is unclear when the transition to mature network patterns occurs.

When studying brain development, individuals are commonly grouped into ‘young’ and ‘old’ cohorts [13,14,15]. In some cases, this boundary is clear, such as when comparing the brains of adults versus children. Functional connectivity-based cut points are less defined during gestation, for example, when comparing brain maturity at mid- and late-fetal stages. The goal of our study was to estimate optimal gestational-age (GA) cut points, or transition periods, where we can subdivide fetal groups based on age-associated changes in network topology and perform meaningful between-group comparisons. In this study, cut points were defined for global functional network metrics that we have recently described; these metrics are normalized clustering and path length, global and local efficiency, modularity, and the small-world index, metrics we have previously described in this fetal cohort [5]. We selected GAs between 28 and 33 weeks as potential cut points based on previous studies reporting transitional electrophysiologic events [16,17] and resting-state studies that demonstrated significant connectivity changes around this period [4,9]. We hypothesized that optimal cut points would likely fall between 30 and 32 weeks coinciding with the dissolution of the transient fetal compartments and consolidation of thalamocortical connections.

## 2. Materials and Methods

### 2.1. Participants

A total of 110 data sets from fetuses of 95 pregnant women with healthy pregnancies were included in the study. RS-fMRI data were collected as part of a prospective, longitudinal study at Children’s National in Washington DC investigating pre- and postnatal brain development in the setting of complex congenital heart disease. We previously described global network topology in this cohort in a recently published study [5]. Conventional T2 for all fetuses revealed structurally normal brains. Pregnant women with known psychiatric/metabolic/genetic disorders, complicated pregnancies (i.e., preeclampsia and gestational diabetes), multiple pregnancies, alcohol and tobacco use, maternal medications, and contraindications to MRI were excluded from the study. For fetuses, those with dysmorphic features on antenatal ultrasound, chromosomal abnormalities by amniocentesis, presentation after 28 weeks’ gestational age, and evidence of congenital infections were excluded.

### 2.2. Acquisition of Resting-State Data

We used a 1.5 T GE MRI scanner (GE Healthcare, Milwaukee, WI) with an 8-channel receiver coil to collect anatomical and resting-state fMRI data. The settings for single-shot fast spin-echo anatomical T2-weighted images (i.e., sagittal, axial, and coronal slices) were as follows: TR= 1100 ms, TE = 160 ms, flip angle = 90°, and slice thickness = 2 mm. The following were the resting-state echo planar images (EPI) scanning parameters: TR = 3000 ms, TE = 60 ms, voxel size = 2.578 mm × 2.578 mm × 3 mm, flip angle = 90°, field of view = 33 cm, matrix size = 128 × 128, and scan duration = 7 min (140 volumes). On average, 5.35 min (25, 75 IQR: 4.6, 6) of resting-state data (107 volumes) were available after preprocessing.

### 2.3. Preprocessing of Resting-State Data

Fetal RS-fMRI data were preprocessed to attenuate the effects of noise on the measured blood oxygen level dependent (BOLD) signals. These steps were previously described in [5,18]. EPI images were slice time corrected, followed by removal of the first four RS volumes to allow for magnetic gradients to stabilize. The data were then oriented to radiologic orientation and despiked. We also performed bias-field correction to address image intensity nonuniformities due to variations in the magnetic field [19]. Next, we corrected for head motion using an algorithm validated in fetuses and newborns [20,21]. Then, RS images underwent intensity normalization to a global mode of 1000 [22]. This was followed by smoothing using an isotropic 4.5 mm full-width half-maximum Gaussian kernel. Band-pass filtering, retaining signals in the range 0.01 Hz–0.1 Hz, and nuisance regression were then simultaneously performed. Along with regression, censoring of high motion frames and volumes with a high number of voxel intensity outliers was performed [23,24]. High motion was defined as frame-by-frame translational and rotational motion >1 mm and >1.5° [7,25], respectively. Frames where more than 10% of voxels had intensities deviating from the voxel time series’ median absolute deviation were also censored from the time series. Regressors included in the general linear model were white matter/CSF signals [26,27,28], linearly detrended rigid motion parameters, and their temporal derivatives [29]. Residual BOLD signals from this regression were analyzed.

### 2.4. Graph Construction

The fetal functional connectome was formed from 200 regions of interest (ROIs, or nodes) (see Supplementary Figure in [5]) defined using a widely used functional clustering technique [30]. In this approach, a normalized-cut spectral algorithm was used to group voxels into nonoverlapping, functionally homogenous ROIs. Clustering was performed using the temporal correlation between voxel BOLD signals, followed by a 2-level clustering approach to create group-level parcellations. As previously described [5], the BOLD signals for each ROI were measured by averaging signals from high-quality voxels that make up each region [31]. Then, all possible pairwise Pearson correlations for all ROIs were computed yielding a 200 × 200 matrix (or 19,900 correlations). The correlation, *r*, between an ROI pair is its functional connectivity. Only significant positive connections (*p_FDR_* < 0.05) were included [32]. In addition, for reliable graph estimates, thresholds were applied such that nodes of individual graphs were at least 95% connected and had average degree k > 2 * ln (number of nodes) [33,34]. Resulting graphs had an edge density range between 0.10 and 0.51. Global network metrics were computed from each fetus’ 200 × 200 binary, undirected graphs at a 0.01 interval. Metrics were averaged across the full density range [35].

### 2.5. Graph Analysis

We used the Brain Connectivity Toolbox (https://sites.google.com/site/bctnet/, accessed on 2 June 2021) to compute normalized (i.e., relative to 100 reference random graphs) values of clustering coefficient (γ) and path length (λ), global efficiency (GE), local efficiency (LE), modularity (Q), and the small-world index (σ) [36,37]. Global metrics related to network segregation and integration were computed. Clustering, local efficiency, and modularity were used to describe network segregation. Normalized path length and global efficiency were used to describe integration. Briefly, the clustering coefficient describes the tendency of neighbors of a node to also be linked to each other. Local efficiency describes the ease of communication among neighbors of one node when that node is removed [38]. Modularity captures the tendency of regions to form densely connected subnetworks while being sparsely linked to other clusters [39]. Characteristic path length is the average shortest distance between two nodes in a network. Global efficiency, the inverse of path length, determines the ability to transmit information across the network. The small-world index describes the balance between segregation and integration commonly found in complex networks such as the brain. A network is considered small-world when σ > 1. For a detailed, mathematical description of these metrics, see [36].

### 2.6. Statistical Analysis

**Figure 1:**
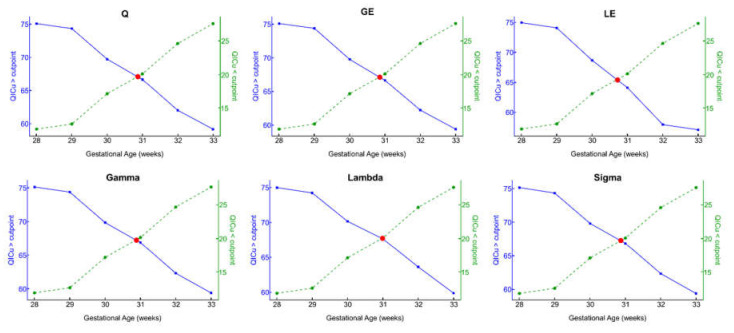
Plot of generalized estimating equation QICu fit indices below (dashed lines) and above (solid lines) the threshold for each metric against gestational age in weeks. Intersecting lines represent the estimated optimal GA binary stratification point.

We modeled the effect of GA at scan on connectivity metrics using separate repeated-measures generalized estimating equations (GEE). A repeated-measures approach was utilized for GEE models, given that 15 patients had two scans. In fetuses with two scans, the mean interval between scans was 7.38 ± 2.81 (mean ± SD) weeks. The interval range was 3–12.86 weeks. Our modeling strategy took three approaches. First, separate overall GEE models per metric were computed with estimated GA slopes at select GA cut points (i.e., 28–33 weeks) calculated. Second, the first approach was repeated, with the participants stratified by gender. Third, the first approach was again repeated with the fetuses grouped into ‘young’ or ‘old’ cohorts based on each GA cut point (e.g., 28 weeks vs. 29+ weeks, ≤ 29 weeks vs. 30+ weeks, etc.), with models stratified by cohort. Lastly, we used the quasi-likelihood independence criterion (QICu) statistic to compare model fit between ‘old’ and ‘young’ cohorts. The QICu is analogous to the Akaike Information Criterion (AIC), and models with a smaller statistic are preferred. Had our data been binary, traditional cutoff analyses would be based on a binary outcome with subsequent use of the Youden’s Index or some other combined measure of sensitivity or specificity. Given that our connectivity outcomes are continuous, we defaulted to the QICu for determining cutoff. The QICu is generally used to distinguish between regression models with a working correlation structure. For consistency, we used an identical correlation structure (exchangeable) across models. Model fit indices such as the -2 loglikelihood (-2LL) utilized in logistic regression are traditionally relied upon to build out or contract models by reducing or increasing parameters. Unlike the -2LL function for binary outcomes data (e.g., logistic regression), the QICu does not utilize a chi-square function that easily translates into statistically significant testing of the addition or subtraction of parameters. There is no currently available way to statistically test the QICu. For this exercise, we are not model building in a traditional way, i.e., adding or subtracting covariates. Each model across each individual GA cut point was identical to avoid issues of parsimony and fitting–we did not want any covariate effects to essentially affect the GA–connectivity relationship. Our model fitting is essentially static. GA cut points were then assessed following graphical depiction (see Figure 1). For each approach, to test the robustness of our GEE connectivity models, GA estimates were evaluated using a bootstrap approach with 1000 iterations (sampling rate = 75%) from which 95% CIs were derived. Confidence intervals were derived using the 2.5 th and 97.5 th percentiles generated from the bootstrapped estimates distribution. For each connectivity metric, we then utilized a graphical approach to compare QICu fit indices for ‘young’ and ‘old’ cohorts at each GA cut point by plotting both groups against each other. All analyses were performed using SAS (ver. 9.4, Cary, NC, USA).

## 3. Results

A total of 110 fetal resting-state scans from 95 healthy fetuses (49 females, 46 males) between 19.14 and 39.71 gestational weeks (median: 34.93; 25, 75 IQR: 31.29, 36.57) were analyzed. All fetuses in the study were eventually born full term, with a median GA of 39.57 (25, 75 IQR: 39.71, 40.29). After rigorous preprocessing, an average of 108 ± 17 volumes were available for analysis. Of these, we only included 80 volumes (i.e., 4 min scan duration) for each participant to reduce bias introduced by variability in time series length. Nevertheless, for this cohort, global metrics for partial time series data closely estimated metrics computed from all available time points (Supplementary Figure S1). Average and maximum frame-by-frame head displacement [24] did not correlate with GA at scan or any of the global network metrics. Demographic and motion findings were previously reported in [5].

### 3.1. GEE Modeling

Trends for normalized clustering and path length, local efficiency, modularity, and the small-world index decreased with increasing GA (Table 1); global efficiency showed an increasing pattern. Age-related trends were consistent for both males and females. Average bootstrapped estimated slopes ranged from β_GA_ = 0.01 (GE) to β_GA_ = 0.22 (σ). Average change in bootstrapped estimated slope from 28 to 33 weeks GA ranged from 3.52% to 17.81%. Among female fetuses (see Supplementary Table S1), following bootstrapping, average bootstrapped estimated slopes ranged from β_GA_ = 0.01 (GE) to β_GA_ = 0.30 (γ). Average change in bootstrapped estimated slope (GA = 28 to GA 33) ranged from 3.54% to 17.92%, with the least change observed for λ (3.78% to 17.87%) and greatest change, GE (3.08% to 18.46%.). Among male fetuses (see Supplementary Table S2), following bootstrapping, bootstrapped estimated slopes ranged from β_GA_ = 0.2 (GE) to β_GA_ = 0.15 (Q). Average change in bootstrapped estimated slope (GA = 28 to GA 33) ranged from 3.61% to 17.83% with the least change observed for λ (3.49% to 17.76%) and greatest change, GE (3.80% to 17.72%.)

### 3.2. Optimal GA Cut Point

For each GA cut point, we then stratified the cohort into ‘young’ or ‘old’ groups based on values below or above the GA threshold of interest (Table 2) and subsequently separately modeled the GA on metric association. The proportion of fetuses allocated to the ‘younger’ cohort based on GA ranged from 11.8% (GA = 28) to 30.9% (GA = 33). Across all metrics, among fetuses classified to the ‘younger’ cohort, the percent change in estimated GA slope ranged from a low of -9.84% (Q) to a high of -39.21% (σ). Lastly, we present model performance QICu metrics (Table 3, Figure 1) for each metric stratified by younger and older cohorts for each GA cut point. For each metric, at each GA cut point, we compared younger and older QICu scores using a modified Youden index to determine the optimal cut point. Optimal cut points fell within the range of 30–31 weeks for all metrics. Supplementary Figure S2 shows global metrics relative to this cut point.

## 4. Discussion

We statistically defined optimal gestational-age cut points that can be used to dichotomize human fetuses into ‘young’ and ‘old’ subgroups based on resting-state functional connectivity. Most network measures’ trends, except for global efficiency, decreased with advancing gestational age. Statistically, optimal GA cut points for small-world index, normalized clustering and path length, global and local efficiency, and modularity were at 30–31 weeks, coinciding with transitional physiologic events that have been shown to be critical to the development of fetal neural circuitry.

That network organization evolves during gestation is not surprising, given how rapidly the brain changes in the third trimester [40]. For example, reduced network segregation with advancing fetal gestational age, demonstrated by decreasing modularity, normalized clustering, and local efficiency, has previously been reported [5,9]. In our previous work, we focused on identifying trends in global network metrics across gestation; here, we focused on identifying transition points where we could meaningfully split fetal cohorts when examining age-related changes in the fetal connectome. The late second to early third trimester of gestation marks a transition in the neural circuitry of the fetal brain, from immature, autonomously generated neuronal bursts to a more sensory-driven pattern [16,41]. The neurobiological underpinnings of the identified inflection points in the network metrics are undetermined, but we speculate that the reported changes in the fetal connectome may be related to maturing electrical patterns. Before 30–32 weeks, electrical activity in the brain is centered around the subplate [42,43,44]. During the midfetal period, or around 15 weeks GA, transient fetal compartments essential to the formation of the fetal neural circuitry have already been formed. The subplate, specifically, serves as a waiting area where postmigratory neurons temporarily connect with subcortical afferents [45]. The establishment of these neural circuits has been linked to the emergence of spontaneous activity transients (SATs), oscillatory neuronal activity that predates evoked brain responses, on electroencephalography recordings in extremely premature infants [46,47]. At around 28–30 weeks, with subcortical afferents synapsing with the cortical plate and refinement of these developing circuits, there is eventual dissolution of the subplate. Electrical activity gradually shifts to the more permanent thalamocortical (subcortical–cortical plate) circuitry, and SATs are replaced by complex cortical EEG patterns [46,48]. The histologic and electrical events described coincide with the identified cut points providing a potential substrate for the transitions seen in the fetal connectome at around 30–31 weeks.

The link between physiologic events and age-related changes in the connectome is currently speculative, but a previous study by Jakab and colleagues [4], where they examined resting-state connectivity in 32 healthy fetuses, also emphasized the relevance of this period. They showed that functional connectivity rapidly increased between 24 and 31 weeks’ gestation and gradually stabilized thereafter, likely due to the third-trimester consolidation of thalamocortical connections.

It is important to point out the limitations of our study. First, we had few late second- to early third-trimester fetuses in our group. A uniformly distributed sample across gestational weeks would enable more robust statistical testing of optimal cut points. Nevertheless, we based our transition points on more than 100 resting-state data sets, a substantially large sample that we think provide reasonable baseline cut points for future investigations. Second, the coupling between the BOLD signal and neural activity, especially in utero, is poorly understood [49]. More studies are needed to elucidate the neural mechanisms underlying the relationship between gestational age, BOLD signal changes, and global network topology. A methodological consideration that bears mention is node selection. Here, we opted to use a functional clustering algorithm to define brain regions. Studies have suggested that global and intermediate metrics are stable across different parcellation strategies, but future studies that systematically test the influence of atlas choice on cut points would help refine our current estimates. In-scanner fetal head motion remains a challenging issue in MRI, and excessive head motion led to the exclusion of a substantial number of otherwise healthy fetuses from our study cohort. To minimize the effect of motion on functional connectivity, we performed a rigorous visual quality assessment, used motion correction algorithms optimized for fetuses, and censored high motion volumes from the hemodynamic time series. Of note, and as previously reported, we did not observe any association between the metrics reported in this study and head motion and gestational age at scan [5]. Lastly, here, we only focused on identifying transition points in global network features which may be related to brain maturation. We did not address whether global network metrics changed in a linear fashion throughout gestation [5,9], an important avenue to explore in future studies.

## 5. Conclusions

We identified GA cut points based on global network topology that could potentially be used for future analyses comparing ‘young’ and ‘old’ fetuses. The period between 30 and 31 weeks coincides with a period in neural circuitry formation where spontaneous activity of early circuit transitions to sensory-driven activity. Additional studies are needed to understand the mechanisms underlying the association between gestational age, neural circuitry changes, and global network topology.

## Figures and Tables

**Table 1:** Overall modeled bootstrapped estimates and 95% confidence intervals at select gestational-age cut points.

Metric	GA at Scan = 28	GA at Scan = 29	GA at Scan = 30	GA at Scan = 31	GA at Scan = 32	GA at Scan = 33
Q	−0.12	−0.1243	−0.1286	−0.1328	−0.1371	−0.1414
	(−0.3497–0.0477)	(−0.3622−0.0494)	(−0.3747−0.0511)	(−0.3871−0.0528)	(−0.3996−0.0545)	(−0.4121−0.0562)
GE	0.0056	0.0058	0.006	0.0062	0.0064	0.0066
	(−0.0102−0.0196)	(−0.0105−0.0203)	(−0.0109−0.0210)	(−0.0113−0.0217)	(−0.0116−0.0224)	(−0.0120−0.0231)
LE	−0.0179	−0.0185	−0.0192	−0.0198	−0.0205	−0.0211
	(−0.0466−0.0315)	(−0.0483−0.0326)	(−0.0500−0.0337)	(−0.0516−0.0349)	(−0.0533−0.0360)	(−0.0550−0.0371)
γ	−0.2449	−0.2537	−0.2624	−0.2712	−0.2799	−0.2887
	(−0.5488−0.4955)	(−0.5684−0.5132)	(−0.5880−0.5309)	(−0.6076−0.5486)	(−0.6272−0.5662)	(−0.6468−0.5839)
λ	−0.0283	−0.0293	−0.0303	−0.0313	−0.0323	−0.0333
	(−0.0788−0.0447)	(−0.0816−0.0463)	(−0.0845−0.0479)	(−0.0873−0.0495)	(−0.0901−0.0511)	(−0.0929−0.0527)
σ	−0.2027	−0.2099	−0.2171	−0.2244	−0.2316	−0.2389
	(−0.4537−0.3698)	(−0.4699−0.3830)	(−0.4861−0.3962)	(−0.5023−0.4095)	(−0.5185−0.4227	(−0.5347−0.4359)

**Table 2:** Stratified modeled bootstrapped estimates and 95% confidence intervals at select gestational-age cut points.

Metric	Exceed	GA at Scan = 28	GA at Scan = 29	GA at Scan = 30	GA at Scan = 31	GA at Scan = 32	GA at Scan = 33
	Cut Point	N = 13/97	N = 14/96	N = 20/90	N = 24/86	N = 30/80	N = 34/76
Q	No	−0.0061	−0.0064	−0.0048	−0.0063	−0.0048	−0.0055
		(−0.0144–−0.0010)	(−0.0139–−0.0017)	(−0.0095− −0.0011)	−0.0072	(−0.0077–−0.0024)	(−0.0082–−0.0031)
	Yes	−0.0014	−0.0014	0.0013	0.0008	0.0027	0.0019
		(−0.0120–0.0075)	(−0.0120–0.0075)	(−0.0066–0.0088)	(−0.0036–0.0203)	(−0.0033–0.0204)	(−0.0047–0.0204)
GE	No	0.0005	0.0006	0.0004	0.0007	0.0008	0.0007
		(−0.0015–0.0032)	(−0.0012–0.0030)	(−0.0007–0.0018)	(−0.0003–0.0018)	(0.0000–0.0016)	(0.0001–0.0015)
	Yes	−0.0001	−0.0001	−0.0004	−0.0006	−0.0003	−0.0002
		(−0.0010–0.0005)	(−0.0010–0.0005)	(−0.0020–0.0004)	−0.0027–0.0002)	(−0.0010–0.0005)	(−0.0011–0.0007)
LE	No	−0.0016	−0.0019	−0.0015	−0.0019	−0.0017	−0.0018
		(−0.0060–0.0018)	(−0.0059–0.0012	(−0.0041–0.0006)	(−0.0041–−0.0001)	−0.0033–−0.0002)	(−0.0031–−0.0005)
	Yes	0	−0.0001	0.0001	0.0006	0.0003	0
		(−0.0016–0.0020)	(−0.0016–0.0020)	(−0.0026–0.0031)	(−0.0008–0.0031)	(−0.0012–0.0015)	(−0.0057–0.0014)
γ	No	−0.0312	−0.0348	−0.0243	−0.0263	−0.0176	−0.0208
		(−0.0510–−0.0149)	(−0.0543–−0.0172)	(−0.0406–−0.0064)	(−0.0386–−0.0134)	(−0.0266–−0.0054)	−0.0298–−0.0083)
	Yes	0.0048	0.0041	0.0021	0.0056	0.0144	0.012
		(−0.0111–0.0341)	(−0.0128–0.0341)	(−0.0102–0.0384)	(−0.0161–0.1078)	(−0.0147–0.1081)	(−0.0167–0.1082)
λ	No	−0.0044	−0.0044	−0.0026	−0.003	−0.003	−0.0029
		(−0.0130–0.0022)	(−0.0123–0.0016)	(−0.0079–0.0021)	(−0.0075–0.0009)	(−0.0064–0.0001)	(−0.0056–−0.0001)
	Yes	−0.0004	−0.0005	0.0004	0.0007	−0.0005	−0.0009
		−0.0036–0.0023)	(−0.0038–0.0022)	(−0.0019–0.0060)	(−0.0020–0.0085)	(−0.0021–0.0015)	(−0.0028–0.0015)
σ	No	−0.0281	−0.0295	−0.0186	−0.0188	−0.0102	−0.0128
		(−0.0492–−0.0139)	(−0.0484–−0.0163)	(−0.0296–−0.0072)	(−0.0265–− 0.0106)	(−0.0166–−0.0000)	(−0.0202–−0.0035)
	Yes	0.0006	−0.0007	−0.0015	0.001	0.0103	0.0079
		(−0.0148–0.0344)	(−0.0167–0.0344)	(−0.0153–0.0344)	(−0.0199–0.1047)	(−0.0177–0.1049)	(−0.0231–0.1050)

**Table 3:** Generalized estimating equation fit indices for stratified modeled bootstrapped estimates and 95% confidence intervals at select gestational-age cut points.

Metric	Exceed	GA at Scan = 28	GA at Scan = 29	GA at Scan = 30	GA at Scan = 31	GA at Scan = 32	GA at Scan = 33
	Cut Point	N = 13/97	N = 14/96	N = 20/90	N = 24/86	N = 30/80	N = 34/76
Q	No	11.867	12.634	17.145	20.132	24.664	27.635
		(9.0000−14.0000)	(9.5000−15.0000)	(14.0000−20.0000)	(16.0000−24.0000)	(21.0000−28.0000)	(23.0000−32.0000)
	Yes	75.1168	74.3619	69.7688	66.7001	62.0553	59.2125
		(72.0004−78.0001)	(74.0000−76.5054)	(66.0000−73.0002)	(62.0002−71.0000)	(57.0405−66.0000)	(55.0000−63.0004)
GE	No	11.867	12.634	17.145	20.132	24.664	27.635
		(9.0000−14.0000)	(9.5000−15.0000)	(14.0000−20.0000)	(16.0000−24.0000)	(21.0000−28.0000)	23.0000−32.0000)
	Yes	75.1168	74.4058	69.7883	66.6606	62.2288	59.4004
		(72.0014−78.0001)	(71.0013−78.0000)	(65.5393−74.0000)	(62.0000−71.0000)	(57.9407−67.0256)	(55.0000−64.5115)
LE	No	11.867	12.634	17.145	20.132	24.664	27.635
		(9.0000−14.0000)	(9.5000−15.0000)	(14.0000−20.0000)	(16.0000−24.0000)	(21.0000−28.0000)	(23.0000−32.0000)
	Yes	74.9755	74.1004	68.6799	64.0572	57.8721	57.0111
		(72.0000−78.0037)	(71.10003−78.0000)	(26.1065−74.0000)	(25.8099−71.0012)	(26.0766−71.9152)	(29.0706−95.2327)
γ	No	11.867	12.634	17.145	20.132	24.664	27.635
		(98.0000−14.0000)	(9.5000−15.0000)	(14.0000−20.0000)	(16.0000−24.0000)	(21.0000−28.0000)	(23.0000−32.0000)
	Yes	75.1457	74.3887	69.8371	66.8617	62.2667	59.3355
		(72.0003−78.0002)	(71.0000−78.0000)	(66.0000−73.0003)	(63.0000−71.0000)	(58.1335−66.0000)	(55.0000−63.4042)
λ	No	11.867	12.634	17.145	20.132	24.664	27.635
		(9.0000−14.0000)	(9.5000−15.0000)	(14.0000−20.0000)	(16.0000−24.0000)	(21.0000−28.0000)	(23.0000−32.0000)
	Yes	74.9952	74.228	70.154	67.6423	63.6002	59.8447
		(72.0004−78.0003)	(71.0007−77.0000)	(65.9967−74.0195)	(62.0000−74.8341)	(57.6592−69.9663)	(55.0000−64.0018)
σ	No	11.867	12.634	17.145	20.132	24.664	27.635
		(9.0000−14.0000)	(9.5000−15.0000)	(14.0000−20.0000)	(16.0000−24.0000)	(21.0000−28.0000)	(23.0000−32.0000)
	Yes	75.2052	74.3849	69.8515	66.8673	62.3361	59.3695
		(72.0002−78.0002)	(71.5005−78.0000)	(66.4253−73.0002)	(63.0000−71.0000)	(59.0000−66.0004)	(55.0000−64.0000)

## Data Availability

The data presented in this study are available upon reasonable request from the corresponding author. The data are not publicly available due to privacy restrictions.

## Supplementary Materials

The following are available online at https://www.mdpi.com/article/10.3390/brainsci11070921/s1 Table S1: Female stratified modeled bootstrapped estimates and 95% confidence intervals at select gestational age-cut points, Table S2: Male stratified modeled bootstrapped estimates and 95% confidence intervals at select gestational-age cut points. Figure S1: Correlation network metrics computed from full (blue) vs. partial (red) time series data. Figure S2: Global network metrics and gestational cut points.

## References

[1] Biswal B., Yetkin F.Z., Haughton V.M., Hyde J.S. (1995). Functional connectivity in the motor cortex of resting human brain using echo-planar mri. Magn. Reson. Med..

[2] Raichle M.E. (2015). The restless brain: How intrinsic activity organizes brain function. Philos. Trans. R. Soc. B Biol. Sci..

[3] Schöpf V., Kasprian G., Brugger P.C., Prayer D. (2012). Watching the fetal brain at “rest”. Int. J. Dev. Neurosci..

[4] Jakab A., Schwartz E., Kasprian G., Gruber G.M., Prayer D., Schöpf V., Langs G. (2014). Fetal functional imaging portrays heterogeneous development of emerging human brain networks. Front. Hum. Neurosci..

[5] De Asis-Cruz J., Andersen N., Kapse K., Khrisnamurthy D., Quistorff J., Lopez C., Vezina G., Limperopoulos C. (2021). Global Network Organization of the Fetal Functional Connectome. Cereb. Cortex.

[6] Doria V., Beckmann C.F., Arichi T., Merchant N., Groppo M., Turkheimer F., Counsell S., Murgasova M., Aljabar P., Nunes R. (2010). Emergence of resting state networks in the preterm human brain. Proc. Natl. Acad. Sci. USA.

[7] Thomason M.E., Dassanayake M.T., Shen S., Katkuri Y., Alexis M., Anderson A.L., Yeo L., Mody S., Hernandez-Andrade E., Hassan S.S. (2013). Cross-Hemispheric Functional Connectivity in the Human Fetal Brain. Sci. Transl. Med..

[8] De Asis-Cruz J., Kapse K., Basu S., Said M., Scheinost D., Murnick J., Chang T., du Plessis A., Limperopoulos C. (2020). Functional brain connectivity in ex utero premature infants compared to in utero fetuses. NeuroImage.

[9] Thomason M.E., Grove L.E., Lozon T.A., Vila A.M., Ye Y., Nye M.J., Manning J.H., Pappas A., Hernandez-Andrade E., Yeo L. (2014). Age-related increases in long-range connectivity in fetal functional neural connectivity networks in utero. Dev. Cogn. Neurosci..

[10] Bullmore E.T., Sporns O. (2009). Complex brain networks: Graph theoretical analysis of structural and functional systems. Nat. Rev. Neurosci..

[11] Rubinov M., Sporns O., Van Leeuwen C., Breakspear M. (2009). Symbiotic relationship between brain structure and dynamics. BMC Neurosci..

[12] Thomason M.E., Brown J.A., Dassanayake M.T., Shastri R., Marusak H.A., Hernandez-Andrade E., Yeo L., Mody S., Berman S., Hassan S.S. (2014). Intrinsic Functional Brain Architecture Derived from Graph Theoretical Analysis in the Human Fetus. PLoS ONE.

[13] Turk E., van den Heuvel M.I., Benders M.J., De Heus R., Franx A., Manning J.H., Hect J.L., Hernandez-Andrade E., Hassan S.S., Romero R. (2019). Functional Connectome of the Fetal Brain. J. Neurosci..

[14] Dosenbach N.U.F., Nardos B., Cohen A., Fair D.A., Power J.D., Church J., Nelson S.M., Wig G.S., Vogel A.C., Lessov-Schlaggar C.N. (2010). Prediction of Individual Brain Maturity Using fMRI. Science.

[15] Van den Heuvel M.P., Kersbergen K.J., De Reus M.A., Keunen K., Kahn R.S., Groenendaal F., De Vries L.S., Benders M.J. (2014). The Neonatal Connectome During Preterm Brain Development. Cereb. Cortex.

[16] Keunen K., Counsell S., Benders M.J. (2017). The emergence of functional architecture during early brain development. NeuroImage.

[17] Vasung L., Turk E.A., Ferradal S., Sutin J., Stout J.N., Ahtam B., Lin P.-Y., Grant P.E. (2019). Exploring early human brain development with structural and physiological neuroimaging. NeuroImage.

[18] De Asis-Cruz J., Krishnamurthy D., Zhao L., Kapse K., Vezina G., Andescavage N., Quistorff J., Lopez C., Limperopoulos C. (2020). Association of Prenatal Maternal Anxiety With Fetal Regional Brain Connectivity. JAMA Netw. Open.

[19] Tustison N.J., Avants B.B., Cook P.A., Zheng Y., Egan A., Yushkevich P.A., Gee J.C. (2010). N4ITK: Improved N3 Bias Correction. IEEE Trans. Med Imaging.

[20] Joshi A., Scheinost D., Okuda H., Belhachemi D., Murphy I., Staib L., Papademetris X. (2011). Unified Framework for Development, Deployment and Robust Testing of Neuroimaging Algorithms. Neuroinformatics.

[21] Scheinost D., Onofrey J., Kwon S.H., Cross S.N., Sze G., Ment L.R., Papademetris X. A fetal fMRI specific motion correction algorithm using 2nd order edge features. Proceedings of the 2018 IEEE 15th International Symposium on Biomedical Imaging (ISBI 2018).

[22] Ojemann J.G., Akbudak E., Snyder A.Z., McKinstry R.C., Raichle M.E., Conturo T.E. (1997). Anatomic Localization and Quantitative Analysis of Gradient Refocused Echo-Planar fMRI Susceptibility Artifacts. NeuroImage.

[23] Cox R. (1996). AFNI: Software for Analysis and Visualization of Functional Magnetic Resonance Neuroimages. Comput. Biomed. Res..

[24] Power J.D., Barnes K.A., Snyder A.Z., Schlaggar B.L., Petersen S.E. (2012). Spurious but systematic correlations in functional connectivity MRI networks arise from subject motion. NeuroImage.

[25] Wheelock M., Hect J., Hernandez-Andrade E., Hassan S., Romero R., Eggebrecht A., Thomason M. (2019). Sex differences in functional connectivity during fetal brain development. Dev. Cogn. Neurosci..

[26] Behzadi Y., Restom K., Liau J., Liu T.T. (2007). A component based noise correction method (CompCor) for BOLD and perfusion based fMRI. NeuroImage.

[27] Muschelli J., Nebel M.B., Caffo B.S., Barber A., Pekar J., Mostofsky S.H. (2014). Reduction of motion-related artifacts in resting state fMRI using aCompCor. NeuroImage.

[28] Jo H.J., Gotts S.J., Reynolds R.C., Bandettini P.A., Martin A., Cox R., Saad Z.S. (2013). Effective Preprocessing Procedures Virtually Eliminate Distance-Dependent Motion Artifacts in Resting State FMRI. J. Appl. Math..

[29] Friston K.J., Williams S., Howard R., Frackowiak R., Turner R. (1996). Movement-Related effects in fMRI time-series. Magn. Reson. Med..

[30] Craddock R.C., James G.A., Holtzheimer P.E., Hu X.P., Mayberg H.S. (2012). A whole brain fMRI atlas generated via spatially constrained spectral clustering. Hum. Brain Mapp..

[31] Peer M., Abboud S., Hertz U., Amedi A., Arzy S. (2016). Intensity-based masking: A tool to improve functional connectivity results of resting-state fMRI. Hum. Brain Mapp..

[32] De Asis-Cruz J., Donofrio M.T., Vezina G., Limperopoulos C. (2018). Aberrant brain functional connectivity in newborns with congenital heart disease before cardiac surgery. NeuroImage Clin..

[33] Achard S., Salvador R., Whitcher B., Suckling J., Bullmore E. (2006). A resilient, low-frequency, small-world human brain functional network with highly connected association cortical hubs. J. Neurosci..

[34] Bassett D.S., Meyer-Lindenberg A., Achard S., Duke T., Bullmore E. (2006). Adaptive reconfiguration of fractal small-world human brain functional networks. Proc. Natl. Acad. Sci. USA.

[35] Lynall M.-E., Bassett D.S., Kerwin R., McKenna P., Kitzbichler M., Muller U., Bullmore E. (2010). Functional Connectivity and Brain Networks in Schizophrenia. J. Neurosci..

[36] Rubinov M., Sporns O. (2010). Complex network measures of brain connectivity: Uses and interpretations. NeuroImage.

[37] Humphries M.D., Gurney K. (2008). Network ‘Small-World-Ness’: A Quantitative Method for Determining Canonical Network Equivalence. PLoS ONE.

[38] Crucitti P., Latora V., Marchiori M., Rapisarda A. (2003). Efficiency of scale-free networks: Error and attack tolerance. Phys. A Stat. Mech. Its Appl..

[39] Newman M.E.J. (2006). Modularity and community structure in networks. Proc. Natl. Acad. Sci. USA.

[40] Stiles J., Jernigan T.L. (2010). The Basics of Brain Development. Neuropsychol. Rev..

[41] Kostović I., Sedmak G., Judaš M. (2019). Neural histology and neurogenesis of the human fetal and infant brain. NeuroImage.

[42] Hoerder-Suabedissen A., Molnár Z. (2015). Development, evolution and pathology of neocortical subplate neurons. Nat. Rev. Neurosci..

[43] Judaš M., Sedmak G., Kostović I. (2013). The significance of the subplate for evolution and developmental plasticity of the human brain. Front. Hum. Neurosci..

[44] Kostovic I., Rakic P. (1990). Developmental history of the transient subplate zone in the visual and somatosensory cortex of the macaque monkey and human brain. J. Comp. Neurol..

[45] Kostović I., Judaš M. (2015). Embryonic and Fetal Development of the Human Cerebral Cortex. Brain Mapp..

[46] Vanhatalo S., Kaila K., Lagercrantz H., Lagercrantz H., Hanson M.A., Ment L.R., Peebles D.M. (2011). Emergence of spontaneous and evoked electroencephalographic activity in the human brain. The Newborn Brain.

[47] Tolonen M., Palva J.M., Andersson S., Vanhatalo S. (2007). Development of the spontaneous activity transients and ongoing cortical activity in human preterm babies. Neuroscience.

[48] Khazipov R., Luhmann H.J. (2006). Early patterns of electrical activity in the developing cerebral cortex of humans and rodents. Trends Neurosci..

[49] Luhmann H.J., Sinning A., Yang J.-W., Reyes-Puerta V., Stüttgen M.C., Kirischuk S., Kilb W. (2016). Spontaneous neuronal activity in developing neocortical networks: From single cells to large-scale interactions. Front. Neural Circuits.

