# Supplementary material for: Functional Connectivity-Derived Optimal Gestational-Age Cut Points for Fetal Brain Network Maturity
Source: Brain Sci. 2021 Jul 12;11(7):921. doi: 10.3390/brainsci11070921 (PMC8304646; doi:10.3390/brainsci11070921)
Supplement: Supplementary file 1 [file brainsci-11-00921-s001.zip › brainsci-1267760-supplementary.pdf]

Table S1. Female Stratified Modeled Bootstrapped Estimates and 95% Confidence Intervals at select Gestational Age Cut Points

Metric	Estimate	GA at Scan = 28	GA at Scan = 29	GA at Scan = 30	GA at Scan = 31	GA at Scan = 32	GA at Scan = 33
Q	-0.0034<br>(-0.0197 - 0.0058)	-0.0951<br>(-0.5518 - 0.1629)	-0.0985<br>(-0.5715 - 0.1687)	-0.1019<br>(-0.5912 - 0.1746)	-0.1053<br>(-0.6109 - 0.1804)	-0.1087<br>(-0.6306 - 0.1862)	-0.1121<br>(-0.6503 - 0.1920)
GE	-0.0002<br>(-0.0021 - 0.0003)	-0.0065<br>(-0.0580 - 0.0083)	-0.0067<br>(-0.0600 - 0.0086)	-0.007<br>(-0.0621 - 0.0089)	-0.0072<br>(-0.0642 - 0.0092)	-0.0074<br>(-0.0662 - 0.0095)	-0.0077<br>(-0.0683 - 0.0098)
LE	0.0006<br>(-0.0008 - 0.0052)	0.0158<br>(-0.0235 - 0.1466)	0.0164<br>(-0.0243 - 0.1519)	0.0169<br>(-0.0252 - 0.1571)	0.0175<br>(-0.0260 - 0.1624)	0.0181<br>(-0.0268 - 0.1676)	0.0186<br>(-0.0277 - 0.1728)
γ	-0.0098<br>(-0.0139 - 0.0013)	-0.2753<br>(-0.3894 - 0.0370)	-0.2851<br>(-0.4033 - 0.0383)	-0.2949<br>(-0.4172 - 0.0396)	-0.3048<br>(-0.4311 - 0.0409)	-0.3146<br>(-0.4450 - 0.0423)	-0.3244<br>(-0.4589 - 0.0436)
λ	0.001<br>(-0.0010 - 0.0088)	0.0291<br>(-0.0291 - 0.2454)	0.0302<br>(-0.0301 - 0.2541)	0.0312<br>(-0.0311 - 0.2629)	0.0322<br>(-0.0322 - 0.2717)	0.0333<br>(-0.0332 - 0.2804)	0.0343<br>(-0.0342 - 0.2892)
σ	-0.0084<br>(-0.0125 - 0.0009)	-0.2349<br>(-0.3512 - 0.0260)	-0.2432<br>(-0.3638 - 0.0269)	-0.2516<br>(-0.3763 - 0.0279)	-0.26<br>(-0.3888 - 0.0288)	-0.2684<br>(-0.4014 - 0.0297	-0.2768<br>(-0.4139 - 0.0307)

Table S2. Male Stratified Modeled Bootstrapped Estimates and 95% Confidence Intervals at select Gestational Age Cut Points

Metric	Estimate	GA at Scan = 28	GA at Scan = 29	GA at Scan = 30	GA at Scan = 31	GA at Scan = 32	GA at Scan = 33
Q	-0.0048<br>(-0.0120 - 0.0023)	-0.1332<br>(-0.3355 - 0.0634)	-0.1379<br>(-0.3474 - 0.0657)	-0.1427<br>(-0.3594 - 0.0680)	-0.1474<br>(-0.3714 - 0.0702)	-0.1522<br>(-0.3834 - 0.0725)	-0.1569<br>(-0.3954 - 0.0748)
GE	0.0006<br>(-0.0001 - 0.0010)	0.0158<br>(-0.0032 - 0.0285)	0.0164<br>(-0.0034 - 0.0296)	0.0169<br>(-0.0035 - 0.0306)	0.0175<br>(-0.0036 = 0.0316)	0.0181<br>(-0.0037 - 0.0326	0.0186<br>(-0.0038 - 0.0336)
LE	-0.0012<br>(-0.0020 - - 0.0002)	-0.0347<br>(-0.0552 - - 0.0045)	-0.036<br>(-0.0571- - 0.0047)	-0.0372<br>(-0.0591 - - 0.0049)	-0.0385<br>(-0.0611 - - 0.0050)	-0.0397<br>(-0.0630 - - 0.0052)	-0.0409<br>(-0.0650 - - 0.0053)
γ	-0.0025<br>(-0.0268 - 0.0341)	-0.0687<br>(-0.7514 - 0.9551)	-0.0711<br>(-0.7782 - 0.9892)	-0.0736<br>(-0.8051 - 1.0233)	-0.076<br>(-0.8319 - 1.0574)	-0.0785<br>(-0.8587 - 1.0915)	-0.0809<br>(-0.8856 - 1.1256)
λ	-0.0023<br>(-0.0035 - - 0.0004)	-0.0645<br>(-0.0979 - - 0.0119)	-0.0668<br>(-0.1014 - - 0.0124)	-0.0691<br>(-0.1048 - - 0.0128)	-0.0714<br>(-0.1083 - - 0.0132)	-0.0737<br>(-0.1118 - - 0.0136)	-0.076<br>(-0.1153 - - 0.0141)
σ	-0.0023<br>(-0.0343 - 0.0310)	-0.0633<br>(-0.9598 - 0.8668)	-0.0656<br>(-0.9941 - 0.8977)	-0.0678<br>(-1.0283 - 0.9287)	-0.0701<br>(-1.0626 - 0.9596)	-0.0724<br>(-1.0969 - 0.9906)	-0.0746<br>(-1.1312 - 1.0215)

Supplementary Figures

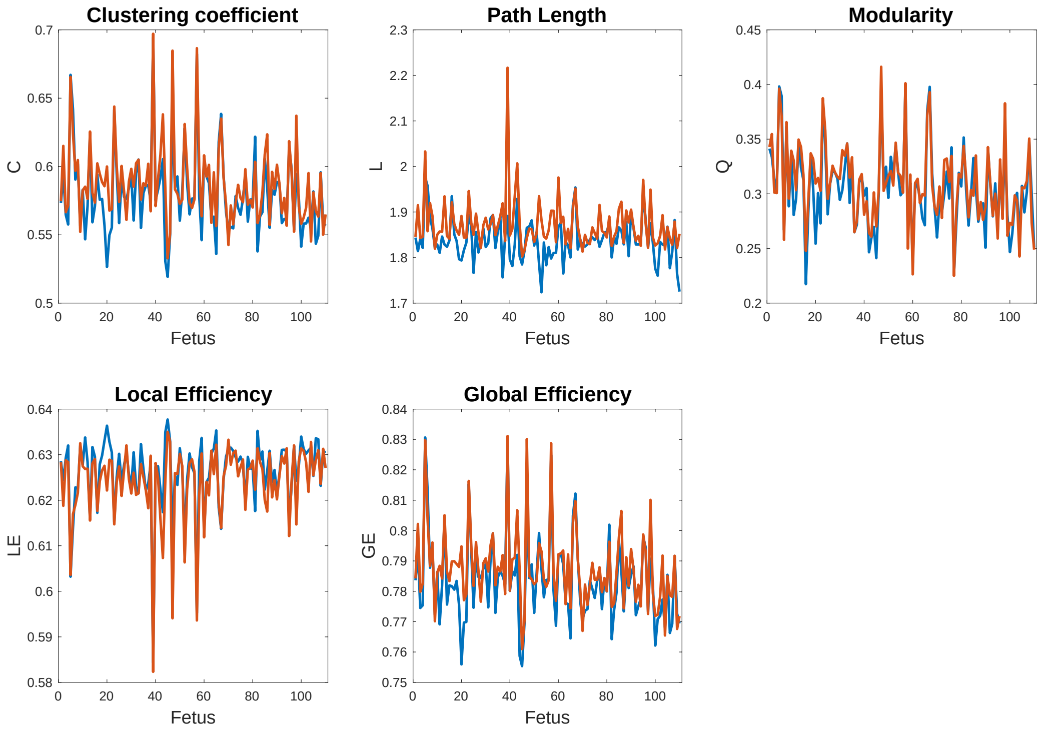

Figure S1. Correlation network metrics computed from full (blue) vs partial (red) time series data.

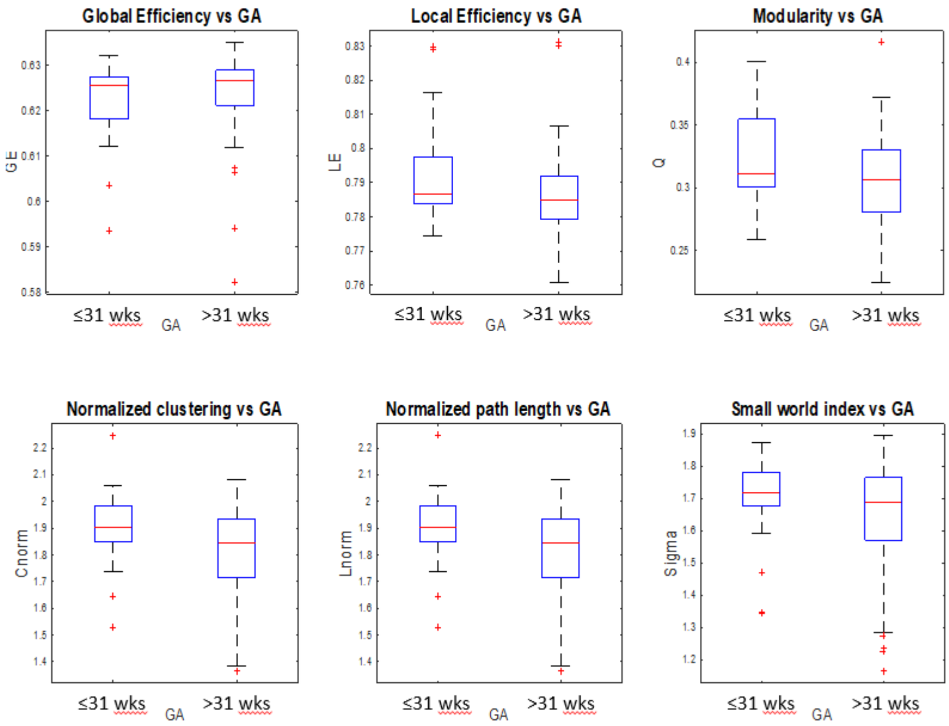

Figure S2. Global network metrics and gestational cut-points.
